# Pleiotropic Effects of Statins in the Light of Non-Alcoholic Fatty Liver Disease and Non-Alcoholic Steatohepatitis

**DOI:** 10.7759/cureus.10446

**Published:** 2020-09-14

**Authors:** Farah Ahsan, Federico Oliveri, Harshit K Goud, Zainab Mehkari, Lubna Mohammed, Moiz Javed, Aldanah Althwanay, Ian H Rutkofsky

**Affiliations:** 1 Internal Medicine, California Institute of Behavioral Neurosciences & Psychology, Fairfield, USA; 2 Cardiology, California Institute of Behavioral Neurosciences & Psychology, Fairfield, USA; 3 Internal Medicine, California Institute of Behavioural Neurosciences & Psychology, Fairfield, USA; 4 Internal Medicine, California Institute of Behavioral Neuroscience & Psychology, Fairfield, USA; 5 Psychiatry, Neuroscience, California Institute of Behavioral Neurosciences & Psychology, Fairfield, USA

**Keywords:** nafld, nash, statins, steatosis, fatty acid oxidation, cytokines, nash and statins

## Abstract

Statins, the lipid-lowering drugs, and non-alcoholic fatty liver disease (NAFLD)/non-alcoholic steatohepatitis (NASH), a lipid-related pathology, share a complex relationship, one known to be hepatotoxic and other being hepatic injury. NASH is an unresolved mystery in terms of treatment. Could statins prove to be a promising solution due to their pleiotropic properties in addition to the cholesterol-lowering effect? This study aims to find statin effectiveness in NAFLD/NASH treatment and prevention of associated adverse outcomes. An extensive data search was done to identify the studies assessing statin effect on NAFLD/NASH and then analyzed to establish the relationship. Several studies demonstrated a reduction in NAFLD/NASH-associated inflammation and fibrosis with statin treatment. These anti-inflammatory and anti-fibrotic effects were through their pleiotropic properties, which were in addition to their cholesterol-lowering effect. In various animal studies, statins were found to improve hepatic lipotoxicity, oxidative stress, inflammatory responses, and fibrosis associated with NASH through multiple pathways. Statins exert these protective effects by recovering the gene expression level of peroxisomal proliferator-activated receptor alpha (PPARα) and therefore restore the mitochondrial and peroxisomal fatty acid oxidation (FAO). Statin treatment also increased the levels of paraoxonase 1 (PON1), an antioxidant and antiatherogenic enzyme that is reduced in NAFLD as well as encounter the hepatic lipotoxicity by resolving cholesterol crystals and Kupffer cells (KCs) with crown-like structures (CLSs). They exhibited antitumor properties by inhibiting proinflammatory cytokines and vascular proliferative factors. Moreover, they restored a healthy liver sinusoidal endothelial cell (LSEC) and hepatic stellate cells (HSC) along with inhibiting the activation of HSC via modulating inducible nitric oxide synthase (iNOS) and expressions of endothelial nitric oxide synthase (eNOS). Besides, they were protective against cardiovascular disease (CVD)-related morbidity and mortality, hepatocellular carcinoma (HCC), and metabolic syndrome (MS) associated with NAFLD/NASH. NASH and its precursor, NAFLD, could be treated and prevented with statins owing to their pleiotropic properties. This study helps to prove this by looking back at different literature and has successfully enlightened the point. Once proved through large clinical trials on humans, it could revolutionize the NASH therapy.

## Introduction and background

Non-alcoholic fatty liver disease (NAFLD), the leading cause of liver disease globally, is becoming the most common indication for a liver transplant due to its progression to non-alcoholic steatohepatitis (NASH). It affects 25% of adults worldwide, 25% of which may progress to NASH [1]. NAFLD is characterized by the accumulation of fat (>5%) in hepatocytes, called hepatic steatosis, without other causes of liver disease like viral hepatitis, increased alcohol consumption, or any chronic liver disease [2]. However, it may progress to NASH or steatohepatitis, which is hepatic steatosis with severe hepatic injury (balloon degeneration of hepatocytes), hepatic inflammation, and fibrosis. Further progression may lead to liver cirrhosis, end-stage liver disease, portal hypertension (PH), and rarely hepatocellular carcinoma (HCC). NAFLD/NASH is also found to be associated with other medical conditions like metabolic syndrome (MS), cardiovascular disease (CVD), diabetes mellitus (DM), and obesity [3]. Being closely linked with insulin resistance (IR) and central obesity, it is regarded as an increased risk for cardiovascular disease, especially atherosclerosis. In fact, NAFLD/NASH has the highest risk of mortality in NAFLD/NASH patients with CVD than liver disease alone.

The development of NAFLD and its progression to NASH is not very well understood. Several mechanisms play a role in liver damage. Different factors that impact the development of hepatic steatosis and its progression to NASH could be presence of free fatty acids (FFA) due to IR and increased peripheral lipolysis, increased synthesis and decreased triglycerides export through very-low-density lipoproteins (VLDL), inflammatory cytokines and adipokines production, hepatic oxidative stress, mitochondrial and peroxisomal dysfunction, and intestinal microbiota (IM) [4]. The diagnosis of NAFLD/NASH is made through features of MS without chronic liver disease and excessive alcohol consumption, imaging studies with evidence of liver steatosis, liver enzymes, fatty liver index, and NAFLD fibrosis score, but it can only be confirmed with liver biopsy [5]. Intrahepatic fibrosis is an established risk factor for cirrhosis, hepatocellular carcinoma, and life-threatening liver failure. However, in NAFLD patients, fibrosis stage is reported to be a superior predictor for overall and liver mortality [6-8].

Currently, the front line management of NAFLD/NASH includes lifestyle modifications with decreased dietary energy intake, weight loss, and exercise. No pharmacological treatment has been approved but the use of insulin-sensitizing agents, lipid-lowering agents, and antioxidants is in practice [9]. There is a need to find a pharma therapy that targets the underlying pathophysiology of this increasingly prevailing disease and can halt its progression to lethal pathologies. Statins could prove to be a strong candidate in that perspective as they are lipid/cholesterol-lowering agents being 3-hydroxy-methylglutaryl coenzyme A (HMG CoA) reductase inhibitors, a rate-limiting enzyme in cholesterol synthesis, and also have pleiotropic effects. Their pleiotropic effects include anti-inflammatory, anti-proliferation, antioxidant, anti-thrombotic, anti-cancer, and immune modulation, hence decreasing inflammation and fibrosis in NAFLD/NASH patients [10]. According to different studies, statins decrease hepatic steatosis by decreasing oxidative stress through increased hepatic antioxidant paraoxonase 1 (PON1) activity [11] and increase mitochondrial and peroxisomal oxidation and induction of peroxisomal proliferator-activated receptor alpha (PPARα), a fatty acid oxidation (FAO) regulator [12]. They also decrease hepatic inflammation by decreasing the expression of proinflammatory cytokines as tumor necrosis factor alpha (TNF-α), interleukin 6 (IL-6), and transforming growth factor beta 1 (TGF-β1) [13]. Moreover, statins improve fibrogenesis in NASH by improving endothelial dysfunction with the restoration of liver sinusoidal endothelial cell (LSEC) and hepatic stellate cells (HSC) phenotype and increase endothelial nitric oxide synthase (eNOS) activity [14-16]. Statins are thought to be protective against HCC as they decrease hepatic expression of angiogenic factors like vascular endothelial growth factor receptor (VEGFR), epidermal growth factor receptor (EGFR), and platelet-derived growth factor (PDGF) [13]. In addition to that, statins also decrease isoprenoid levels [17] and resolve cholesterol crystal, crown-like structures (CLSs) [18] and inactivate hepatic stellate cells (HSC) [19] which prevents inflammation and fibrosis in NAFLD/NASH. In the light of the above findings, statins might play an important role in preventing, delaying, and transforming hepatic steatosis into NASH. Since they are considered hepatotoxic, statins are not prescribed as frequently in a patient with liver disease, but recently several studies have shown they are beneficial and safe in preventing and treating hepatic inflammation and fibrosis associated with NAFLD and NASH [20]. Additionally, statin treatment is reported to decrease the risk of atherosclerotic disease and NAFLD related CVD morbidity and mortality. As a matter of fact, they are the only class of antihyperlipidemic that decreases CVD in NAFLD [21].

**Figure 1:**
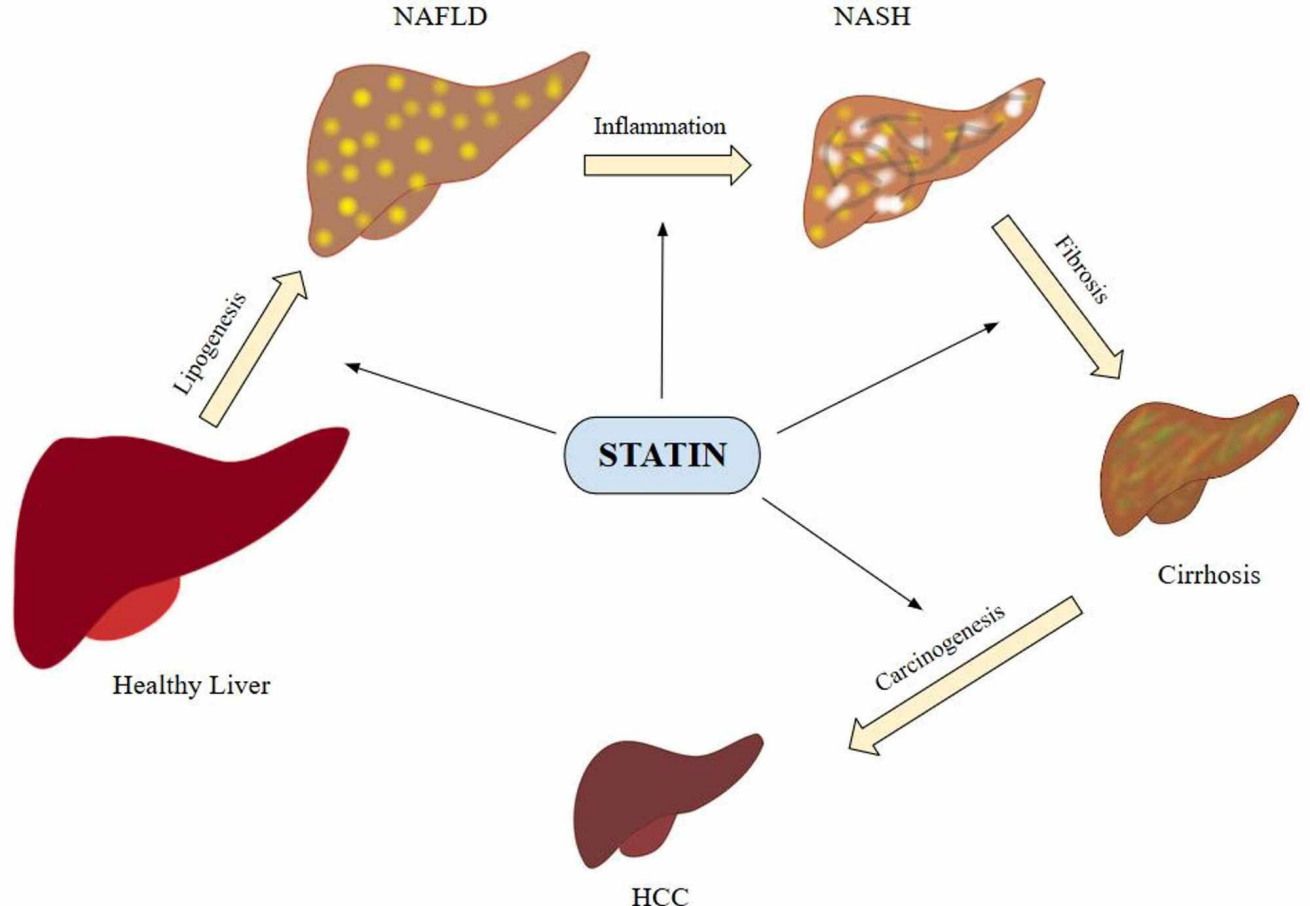
Non-alcoholic fatty liver disease pathogenesis and statins NAFLD - Nonalcoholic fatty liver disease NASH - Non-alcoholic steatohepatitis HCC - Hepatocellular carcinoma

Statins are mainly considered antihyperlipidemic and their pleiotropic properties are not well appreciated. This study aims at reviewing medical literature to discover different pathogenic pathways of NAFLD and its progression to NASH and to find out the effectiveness of statins in such cases (Figure 1). Hopefully, this will help justify more diversified use of statins and subsequently increase awareness of their use in NAFLD and NASH. Since NAFLD/NASH are becoming a growing public health issue therefore to prevent and effectively pretreat, statins use might be a breakthrough as NASH has the highest CVD risk but dyslipidemia remains undertreated.

## Review

Method

Search Sources and Search Strategy

This study was conducted by using PubMed as the main database exclusively and was last searched on June 25, 2020, to find the related articles. Keywords used were NAFLD, NASH, statins, steatosis, fatty acid oxidation, and cytokines. The search with the last 10 years inclusion criteria yielded 18,918 articles with NAFLD, 10,308 articles for NASH, and 30,537 articles for the keyword statins. Then we searched using the combination of keywords to narrow down our data. NASH and statins yielded 112 articles and NASH, statins, and steatosis listed 91 published articles. Combining NASH, statins and fatty acid oxidation just listed 13 articles and NASH, statins, and cytokines came up with 12 articles only. 

Study Selection and Eligibility Criteria

All studies were selected related to the topic of NAFLD/NASH and statins and all unrelated studies were excluded. We just selected the studies which were peer-reviewed and any grey literature was excluded as well. All selected articles were in the English language only. Moreover, no geographical or global considerations were made. The studies were included without the restriction of study type, including investigation of clinical trials, systematic review, and meta-analysis. Age, gender, and ethnicity were also not taken into consideration while selecting the studies.

The articles were selected as full text as well as abstracts. Approximately 112 articles were identified, but only those that reported original study data results with clear methodology conducted in the last 10 years were included. In addition to those, we included studies from references of selected articles, that were from earlier publication dates, which were considered critical to the summary of the effects of statins. From finally selected articles, any non-peer-reviewed and duplicate papers were excluded. That narrowed the data to 50 articles including both full texts as well as abstracts. The focus of the study was on the effects of statins on NAFLD/NASH through different mechanisms, targeting the pathogenesis of the disease. We focused only on those studies that show the effectiveness of statins and the studies of other drugs' effects on NAFLD/NASH were excluded. Moreover, the data was collected ethically and legally following the Preferred Reporting Items for Systematic Reviews and Meta-Analyses (PRISMA) checklist for quality appraisal.

Results

We searched exclusively on PubMed and found the following number of articles with each keyword as mentioned in Table 1.

**Table 1:** No. of research articles for the searched keywords NAFLD - Nonalcoholic fatty liver disease NASH - Non-alcoholic steatohepatitis

Keywords	Database	Number of Results
NAFLD	PubMed	18,918
NASH	PubMed	10,308
statins	PubMed	30,537
NASH and statins	PubMed	112
NASH, statins, steatosis	PubMed	91
NASH, statins, fatty acid oxidation	PubMed	13
non-alcoholic steatohepatitis, statins, cytokines	PubMed	12

Out of all these studies, 112 were the most relevant to find the effect of statins on NAFLD and NASH. From these 112, we found only 41 studies the most inclusive of the effectiveness of statins in NAFLD and NASH, of which 23 were abstracts only and 18 were free full articles. All studies were in English and none were translated. No global or geographical restrictions and no age, race, or gender considerations were made. All studies were peer-reviewed published articles and no grey literature was included. Excluding the studies related to other drugs and duplicate articles brought the number to 33. It was a mixed study including traditional reviews, systematic reviews, clinical trials, meta-analysis, etc.

Discussion

Pathogenesis and Future Molecular Targets

NAFLD, a new epidemic, histologically ranges from simple steatosis to steatohepatitis and then to cirrhosis. The liver pathology starts with fat accumulation in the liver, with possible causes being increased hepatic free fatty acids (FFA), increased synthesis and decreased release of triglyceride (TG) through very-low-density lipoprotein (VLDL), decreased hepatic beta-oxidation and increased liver de novo lipogenesis (DNL). Moreover, NAFLD is usually associated with insulin resistance (IR) that will increase lipolysis from adipose tissue, which in turn increases FFA that is taken up by the liver and causes lipid peroxidation, increasing the production of the pro-inflammatory cytokine. The FFA increase might as well exceed mitochondrial beta-oxidation which results in a further increase in oxidative stress and inflammation. IR also increases the liver DNL by stimulating the enzymes in its pathway resulting in increased TG synthesis but decreased VLDL secretion with resultant TG accumulation in the liver.

NASH or steatohepatitis progression depends on different factors such as FFA, inflammatory cytokines and adipokines, oxidative stress, and mitochondrial dysfunction [3]. FFA after beta and omega-oxidation accumulates reactive oxygen species (ROS) [22]. Proinflammatory cytokines including tumor necrosis factor alpha (TNF-α) and interleukin 6 (IL-6), which are produced by the liver and adipose tissue from nuclear factor kappa B (NF-κB) activation, are usually elevated and also associated with IR. In addition, adiponectin, an anti-inflammatory adipokine produced by adipose tissue, is decreased which further decreases FFA oxidation and hepatic gluconeogenesis. Recently, intestinal microbiota (IM) is suggested to play a role in NAFLD pathogenesis by various pathways [3]. Several animal studies have proved cholesterol to be lipotoxic and promote NASH development by forming cholesterol crystals in steatotic hepatocytes, which activates nucleotide-binding oligomerization domain, leucine-rich repeat and pyrin domain containing protein 3 (NLRP3) inflammasomes and Kupffer cells (KCs) enlargement forming “crown-like structures” (CLSs), and resulting into chronic inflammation and fibrosis [23]. The fibrogenic mechanisms in NASH are complex but include IR, oxidative stress, cytokines specially adipokines. Many studies have shown that an increase in transforming growth factor beta 1 (TGF-β1) in hepatic tissue and serum, activates hepatic stellate cells (HSC), a major source of extracellular matrix (ECM), whose altered deposition can increase fibrosis and hence cirrhosis [19]. All the above studies explained the development of steatohepatitis through different mechanisms with the main cause being excessive fat accumulation in the liver and its strong association with IR which results in FFA availability that increases oxidative stress by proinflammatory cytokines, mitochondrial dysfunction, etc. IM and cholesterol crystallization contributes to chronic inflammation and fibrosis with HSC activation adding up to later. Summarizing all these studies we can say that there is no single pathway for NAFLD/NASH pathogenesis. All the above studies complement each other in that respect.

In the future, NASH is expected to be more common and there is no effective treatment so far. Current research focuses on an agent that can target nuclear transcription factors, lipotoxicity, oxidative stress, metabolic and inflammatory response; an agent that can prevent, resolve, and reverse inflammation and fibrosis in the liver [24]. Multiple therapeutic approaches are considered. Statins could be one of those due to their pleiotropic effects on inflammation and fibrosis, in addition to the antihyperlipidemic effect they are used more commonly for.

Statins Pleiotropy

Statins, 3-hydroxy-methylglutaryl coenzyme A (HMG CoA) reductase inhibitors, are primarily used for primary and secondary prevention of coronary heart disease. Over a while, several studies have shown that they are more than cholesterol biosynthesis inhibitors. They exert effects that are beyond lipid-lowering. These cholesterol independent effects are called "pleiotropic" effects and include anti-inflammatory, antioxidant, antiproliferative, anti-thrombogenic, and immunomodulatory effects with plaque stability and normalization of sympathetic outflow as well [25,26].

Target points for statin effects in NAFLD/NASH are the following:

Statins and Small Guanine Triphosphate Binding Proteins (GTPases)

Statins have been shown to have a protective effect on NAFLD/NASH through inhibition of small GTPases (family of hydrolase enzymes that bind to the nucleotide guanosine triphosphate (GTP) and hydrolyze it to guanosine diphosphate) such as ras homologous family member A (RhoA) and rat sarcoma protein (Ras) by decreasing the isoprenoid levels. Prenylation is an important reaction that allows anchoring of proteins such as small GTPases to cell membranes and their interaction with downstream effectors. Thus, statins modify intracellular signaling of different receptors and therefore, decrease inflammation and fibrosis due to inhibition of RhoA and Ras downstream signaling. The study was performed by Robert Schierwagen et al., on twelve-week-old mice after feeding them with a high-fat, cholesterol-rich diet for seven weeks to develop NASH. The liver biopsies were taken and the effects of different interventions were studied. It is thought that small GTPases like Ras-related C3 botulinum toxin substrate 1 (RAC1), Ras, and RhoA play an important role in the development of metabolic syndrome (MS). Different inhibitors were used to find the role of each GTPase including NSC 23766 trihydrochloride (NSC), a selective inhibitor of RAC1, and *Clostridium sordellii* lethal toxin (LT), that inhibits RAC1 and Ras at the same extent, and lastly simvastatin (SMV), an inhibitor of RAC1, Ras, and RhoA. The study results showed that SMV decreased inflammation and fibrosis through inhibition of Ras extracellular signal‑regulated kinase (Ras/ERK) and RhoA/Rho kinase signaling pathways but RAC1 inhibition had no effect. Moreover, no effect of the small GTPase inhibition was detected on steatosis. Thus simvastatin exhibited anti-inflammatory and antifibrotic effects in NAFLD by inhibition of RhoA- and Ras-mediated pathways without changing steatosis [17].

Statins and Proliferator-Activated Receptor α (PPARα)

Another mechanism by which statins may exert their pleiotropic effect is via increasing the gene expression of peroxisomal proliferator-activated receptor α (PPARα), the master regulator of fatty acid oxidation (FAO) hence improving the peroxisomal and mitochondrial FAO, that is decreased in NASH patients. The mitochondria play an important role in steatohepatitis by the accumulation of cholesterol. Whereas peroxisomes exert their metabolic effect by the degradation of very long chain and branched-chain fatty acids, which cannot be instantly oxidized in mitochondria. Peroxisomes also play a critical role in the maintenance of intracellular redox balance. A study was conducted by Park et al. on seven-week-old mice after giving them normal chow or a methionine- and choline-deficient diet (MCDD) with or without various statins like fluvastatin, pravastatin, simvastatin, atorvastatin, and rosuvastatin (15 mg/kg/day) for six weeks. Histological lesions were analyzed and mitochondrial and peroxisomal FAO were measured in the liver. The results showed an increase in hepatic mitochondrial and peroxisomal FAO via induction of PPARα and its target genes by statins. Furthermore, out of all the statins, fluvastatin was found to have the most effect. Thus the study found that statin treatment decreases both hepatic steatosis and steatohepatitis. This study indicated a possibility that improving peroxisomal and mitochondrial function by statins increases the hepatic FAO which may prevent NASH [12].

Statins and Paraoxonase 1 (PON1) 

Statins increase the levels of paraoxonase 1 (PON1), an antioxidant and antiatherogenic enzyme synthesized mainly in the liver and hydrolyze peroxides and lactones associated with lipoproteins, and is therefore linked to a reduction in oxidative stress and inflammation. It is also associated with high-density lipoproteins (HDLs) in the blood, resulting in an atheroprotective effect. The study by Milaciu et al. was a clinical trial conducted on 50 patients with NAFLD and 20 normal subjects matched for age and sex. NAFLD patients were further divided into two groups (25 patients each), one of which received atorvastatin 40 mg tablet for eight months. The following investigations were performed: abdominal ultrasonography, serum PON1 activity level, liver function tests, serum lipid profile, fasting, and postprandial blood glucose and serum levels of malondialdehyde (MDA) and glutathione peroxidase (GP). Results showed a significant decrease in serum PON1 activity with an increase in MDA and GP activity (i.e significant increase in lipid peroxidation rate) in a non-interventional group but after atorvastatin therapy, there was a significant increase in serum PON1 activity and a significant decrease in serum MDA levels. Therefore decreasing PON1 and increased MDA might be a biochemical marker for lipid peroxidation which could be improved with statins resulting in the prevention of steatohepatitis. Moreover, LM + MM genotypes of the PON1 gene L55M polymorphism were found to be an independent predictor for the presence of NAFLD [22]. So the study proved the link of low PON1 concentrations and LM+MM genotypes of PON1 gene L55M polymorphism with NAFLD while statins improved it.

Statins and Kupffer Cell Crown-Like Structures

Statins help ameliorate hepatic lipotoxicity, a mechanism for NASH development when the liver is exposed to lipotoxic lipid species, by resolving cholesterol crystals and Kupffer cells (KCs) with crown-like structures (CLSs) hence improving inflammation and fibrosis associated with it. Recent experimental studies suggest that free (unesterified) cholesterol (FC) is an important lipotoxic molecule that promotes the development of NASH by enlarged KC surrounding steatotic hepatocytes containing cholesterol crystals and forming characteristic crown-like structures similar to those recently described in inflamed visceral adipose tissue. These enlarged activated KCs then take the appearance of the lipid-laden “foam cells” found in atheroma. Moreover, the cholesterol crystals also appear to activate the NLRP3 inflammasome in animal models of atherosclerosis, therefore providing a mechanism by which exposure of KCs to cholesterol crystals could lead to chronic inflammation and resultant fibrosis in NASH. In a study by Ioannou et al., mice were fed a high-fat (23%) and 0.2% cholesterol-containing diet for 16 weeks and then assigned to four intervention groups for eight weeks: a) vehicle control, b) ezetimibe (5 mg/kg/day), c) atorvastatin (20 mg/kg/day), or d) ezetimibe and atorvastatin. In vehicle-treated mice, the liver developed fibrosing NASH with cholesterol crystallization in lipid droplets extending over 3.3% (SD, 2.2%) of liver surface area. The lipid droplets with cholesterol crystallization were surrounded by TNFα-positive (activated) KCs forming CLSs (≥3 per high-power field). KCs forming CLSs stained positive for NLRP3, showing activation of the NLRP3 inflammasome in response to cholesterol crystals. Whereas, mice treated with ezetimibe and atorvastatin showed almost complete resolution of cholesterol crystals [0.01% (SD, 0.02%) of the surface area] and CLSs (0 per high-power field), and improvement of fibrosis. But ezetimibe or atorvastatin alone had intermediate effects on cholesterol crystallization, CLSs, and NASH [18]. This study indicated how hepatic cholesterol crystals activate the NLRP3 inflammasome within the KCs that form CLSs which is found to be associated with the progression of NASH. Moreover, it stresses the benefits of cholesterol-lowering drugs including statins in improving NAFLD pathology and disease progression by preventing inflammation and fibrosis.

Statins and Profibrogenic Factors 

Statins have anti-inflammatory and anti-fibrogenic effects and can prove to be protective against hepatocellular carcinoma (HCC) associated with advanced stages of NAFLD. Yokohama et al. conducted a study on stelic animal model (STAM) mice after being fed with a high-fat diet (HFD) to develop HCC. The histological analysis of hepatic tissue, as well as blood samples, were obtained to find the effect of rosuvastatin (Ros). The results have shown a decrease in the expression level of proinflammatory cytokines, such as TNF-α, IL-6, interleukin 1 beta (IL-1β), interferon gama (IFN-γ), and profibrogenic factor-like transforming growth factor beta 1 (TGF-β1) as well as angiogenic factors like vascular endothelial growth factor receptor (VEGFR), epidermal growth factor receptor (EGFR) and platelet-derived growth factor (PDGF), by almost 50% in the Ros group. This finding suggests that Ros, a statin, has an antitumor effect mediated by the downregulation of vascular proliferative factors in addition to its antihyperlipidemic effect, therefore protective against HCC associated with NASH [13].

Statins and Liver Sinusoidal Endothelial Cell (LSEC)

Statins improve sinusoidal endothelial dysfunction and prevent portal hypertension (PH). Many studies have verified that clinical complications of NASH are due to PH, which may precede fibrosis development and is caused by liver sinusoidal endothelial cell (LSEC) dedifferentiation/capillarization with decreased nitric oxide (NO) production and subsequent hepatic stellate cells (HSC) activation, resulting in increased intrahepatic vascular resistance in cirrhosis and raising portal pressure (PP). A study was done by Bravo et al. with rats on a high-fat glucose-fructose diet (HFGFD) or a control diet (CD) for eight weeks and then they were treated with simvastatin (sim), atorvastatin (ato), or vehicle. Sinusoidal endothelial dysfunction was assessed in LSEC and HSC from the liver samples on a histological and biochemical basis while hemodynamic changes were also analyzed. HFGFD rats demonstrated full NASH features without fibrosis and increased portal pressure compared with CD rats (10.47 ± 0.37 mmHg vs 8.30 ± 0.22 mmHg; p < 0.001). In addition, HFGFD rats showed a high percentage of capillarized (CD32b−/CD11b−) LSEC (8% vs 1%, p = 0.005), a phenotype associated with HSC activation. Statin treatments resulted in decreased portal pressure (sim: 9.29 ± 0.25 mmHg, p < 0.01; ato: 8.85 ± 0.30 mmHg, p < 0.001), reversed NASH, along with LSEC differentiation recovery and a HSC activation regressed to a more inert phenotype. The study results showed that statins improved NASH histology as well as PH along with recovering sinusoidal endothelial function by restoring a healthy LSEC and HSC phenotype which leads to a decrease in PP and improved prognosis [27]. Therefore, statins are vasoprotective to liver sinusoidal endothelium by maintaining LSEC differentiated phenotype.

Statins and Hepatic Stellate Cells (HSC) Activation 

Statins have anti-inflammatory and anti-oxidative effects that can help ameliorate steatosis-induced hepatic fibrogenesis in NASH. Chong et al. investigated the anti-fibrotic properties of fluvastatin (Flu) in vitro and in vivo. Human hepatoma cell line (HepG2) was grown and primary rat hepatocytes (PRHs) were collected and cultured and treated with Palmitate (PA). Intracellular H2O2 levels for reactive oxygen species (ROS) as well as changes in expressions of nicotinamide adenine dinucleotide phosphate (NADPH) oxidase gp91phox subunit, α-smooth muscle actin (α-SMA), and nuclear factor-kappa (NFκB) p65 nuclear translocation were measured. Moreover, expressions of pro-inflammatory genes, pro-fibrogenic gene, and protein expression of α-SMA were also analyzed in cultured rat hepatic stellate cell (HSC), with or without Flu-pretreatment. In an in vivo study, rats were fed choline-deficient L-amino acid defined (CDAA) diet to induce NASH and then Wistar rats (n = 28) randomly assigned to normal controls (n = 4), CDAA diet with vehicles, and CDAA diet with Flu (5 mg/kg or 10 mg/kg) (n = eight each) for four or eight weeks. After that, livers were histologically analyzed. Results of the study showed that, in vitro, Flu inhibited PA-induced free-radical production, gp91phox expression, and NFκB p65 translocation in HepG2 and PRHs, as well as suppressed α-SMA protein and pro-fibrogenic gene expressions in rat HSC. In vivo, Flu reduced steatosis and fibrosis scores, α-SMA protein expression, mRNA expression of pro-inflammatory and pro-fibrogenic genes in livers of CDAA rats [10].

According to several previous studies, reactive oxygen species (ROS) generation is associated with chronic liver diseases. NADPH oxidase gp91phox subunit, which is present in a variety of hepatic cells including the Kupffer cells, hepatocytes, and HSC, participates in liver inflammation and fibrosis. Moreover, proinflammatory cytokines and NFκB activation are also related to inflammation and resultant activation of HSC. α-SMA protein expression is a marker of HSC activation through the paracrine effect of the hepatocyte. All these processes were suppressed by Flu treatment in the above study. Moreover, Flu inhibits HSC activation which is associated with fibrogenesis, directly as well as through the paracrine effect of hepatocytes. So, this study proved the anti-fibrotic effects of Flu in steatosis-induced hepatocyte damage and showed that Flu decreases ROS production, gp91phox expression, NFκB activity, and pro-inflammatory gene expressions, and reduce α-SMA protein expression and profibrogenic genes expressions in HSC. Thus suppressing inflammation and oxidative stress which in turn reduce steatosis-induced hepatic fibrogenesis [10]. In addition to its lipid-lowering effects, the results of the present study support the role of Flu as an effective anti-fibrosis agent that has the potential to become a promising candidate for NASH therapy.

Another study by Wang et al. demonstrated a simvastatin role in liver fibrosis both in vivo and in vitro. Male Wistar rats were fed a high-fat diet to develop NASH related liver fibrosis and simvastatin (4mg·kg-1·d-1) was given intragastrically until hepatic histological findings confirmed the fibrosis. Human hepatic stellate cell (HSC) line LX-2 cells were cultured and treated with transforming growth factor β1 (TGF-β1), served as a positive control, simvastatin, TGF-β1 plus simvastatin, Nω-nitro-L-arginine methyl ester hydrochloride (L-NAME, an inhibitor of nitric oxide synthase), and L-NAME with simvastatin. The expressions of endothelial nitric oxide synthase (eNOS), inducible nitric oxide synthase (iNOS), and Collagen І, as well as cellular α-SMA, were measured in liver tissue and HSC. The results exhibit an increase in hepatic mRNA and protein expressions of iNOS, α-SMA, and Collagen І and decrease in eNOS during NASH progression. While rats in the simvastatin group had vise versa that is decreased expressions of iNOS, α-SMA, and Collagen І and increased expressions of eNOS. In vitro, simvastatin inhibited LX-2 cell activation due to TGF-β1 or L-NAME by increasing the expression of eNOS and decreasing the expression of iNOS. In conclusion, this study proved the antifibrotic effect of simvastatin by decreasing the hepatic lipid deposition, hepatic inflammation, and the development of fibrosis by inhibiting the activation of HSC via modulating iNOS and eNOS [19].

**Figure 2:**
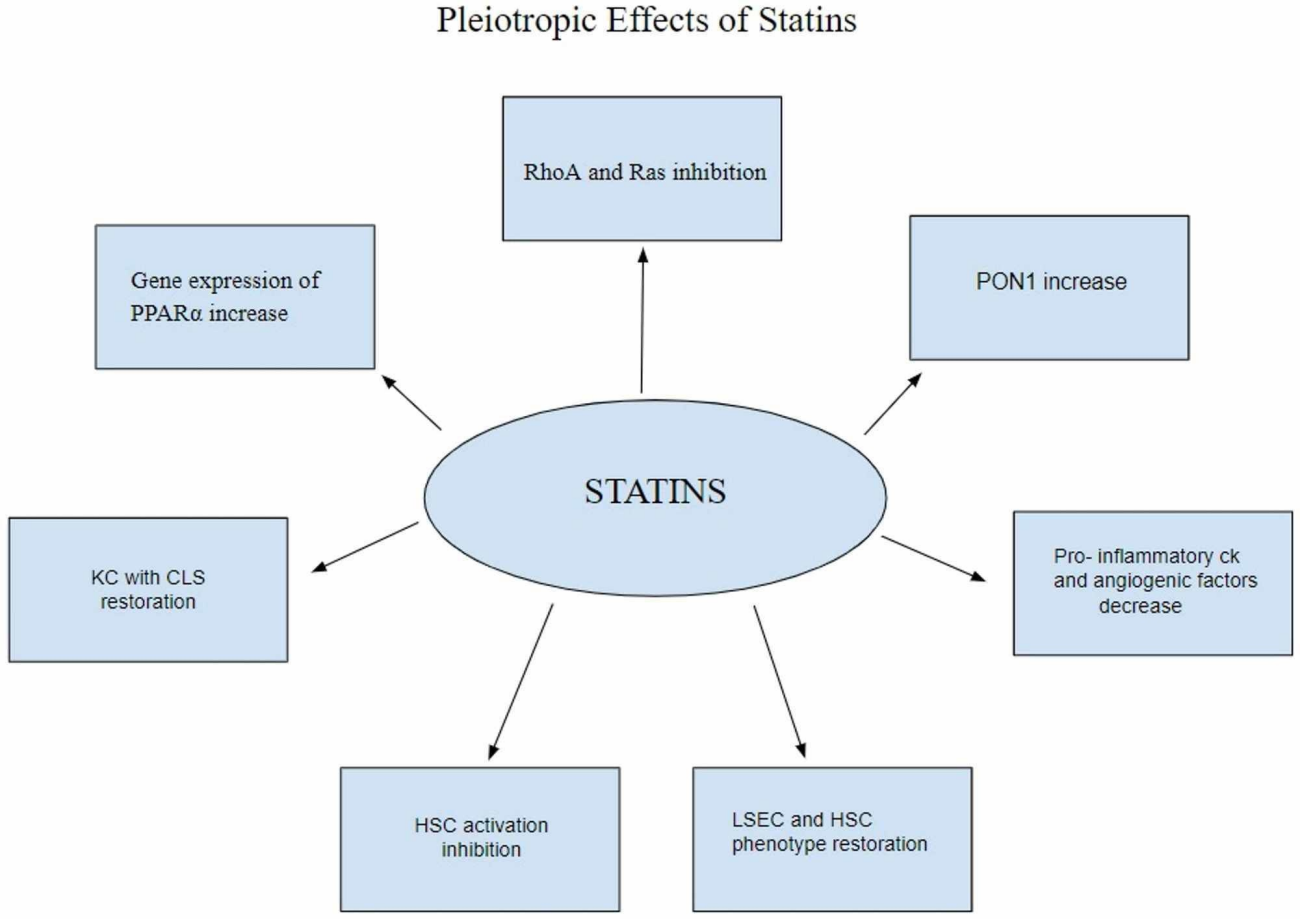
Pleiotropic effects of statins PON1 - Paraoxonase 1, PPARα - Peroxisomal proliferator-activated receptor alpha, LSEC - liver sinusoidal endothelial cell, HSC - Hepatic stellate cells, KCs - Kupffer cells, CLSs - crown-like structures

All the above studies elicited the benefits of statin use in NAFLD/NASH exhibiting different pathways (Figure 2). All were within the last seven years. Most of these were animal studies except the one by Milaciu et al. [22], which is a human study but had several limitations such as not using liver biopsy to establish NAFLD diagnosis as the rest of the studies did, hence need to be proved by larger clinical trials on humans.

Statins in a Protective Role

NAFLD is found to be strongly associated with obesity, dyslipidemia, type 2 diabetes, and metabolic syndrome. It is also associated with CVD, making it the leading cause of mortality in NAFLD. NASH, the more severe form is related to cirrhosis and an increased risk of HCC. Therefore, it is important to identify and proactively treat NAFLD/NASH to prevent all the above-associated risk factors [20]. Currently, no effective treatment for NAFLD/NASH has been approved. According to several studies statins have proven to be effective in treating as well as preventing many risk factors associated with NAFLD/NASH. A narrative review by Athyros et al. assessed the evidence supporting statin use for the treatment of NASH and the reduction of the high CVD risk. The data for animal studies indicated the benefit of statin use in improving liver histology. In humans, post hoc analyses of three randomized controlled trials (n=1,600, n=1,123, n=8,864) found that atorvastatin (even in 80 mg/day) improves the liver enzyme and ultrasonographic findings in NAFLD/NASH. Moreover, statin treatment decreases the CVD morbidity and mortality to half in statin-treated NAFLD/NASH patients. The results of the study show the protective effect of statin treatment on steatosis, steatohepatitis, and fibrosis and improve or cure NAFLD/NASH, as well as significantly reduce CVD morbidity and mortality [28]. Another literature review by Doumas et al. analyzed the statin effect on NAFLD and NASH. After reviewing three post hoc clinical trials it concluded that statins (specifical atorvastatin) reduce CVD as much as twice than those with normal liver function. Moreover, statin treatment may also be protective against HCC related to NAFLD/NASH [29]. In a randomized clinical trial by Kargiotis et al., 20 patients with metabolic syndrome (MS) and biopsy-proven NASH were treated with rosuvastatin (10 mg/d) monotherapy for one year and followed up with repeat liver biopsy and ultrasonography. The results showed complete resolution of NASH and normalization of liver enzymes, lipid profile, serum uric acid (SUA), and blood glucose levels in 19 patients whereas no patient had MS at the end of the study, suggesting that rosuvastatin monotherapy could improve NASH and completely resolves MS within 12 months. The study results further strengthen the role of statins in NAFLD/NASH treatment and thier effectiveness against the reduction of the associated risk factors like MS [30].

We reviewed four studies to find statin effectiveness in improving the risk factors. Three of these were narrative reviews based on previous clinical trials with large samples, hence can be considered powerful. One was a randomized controlled trial but with a small sample size (20 patients) therefore, results need to be confirmed with larger prospective studies. The last study also lacked a control group because based on lipid guidelines it was unethical to deprive statin treatment in NASH patients with MS.

Statins and Liver Safety 

Statins have always been a safety concern for NAFLD/NASH patients as they are considered hepatotoxic. NAFLD has higher CVD risk but statins are not often prescribed especially in patients with high plasma aminotransferase levels. Therefore, patients are deprived of an effective and most needed therapy which could be preventing future complications and may reverse the present pathology. To evaluate the safety of statins, a randomized controlled trial was done by Bril et al. including 101 patients with biopsy-proven NASH and prediabetes/T2DM were followed for up to 36 months, out of which 86 were receiving statins. The patients were assessed for biochemical and histological changes. Results showed no changes in liver histology or hepatic insulin resistance in patients with NASH, newly started on a statin, or receiving a placebo during the study. The study concluded that statins can be safely used in patients with NASH and not be denied to these patients, who already have a very high risk for cardiovascular disease [31]. A meta-analysis by Eslami et al. concluded that statins may improve serum aminotransferase levels as well as ultrasound findings in NASH patients [32]. According to other review studies by Athyros et al. [28] and Doumas et al. [29], statin administration is an effective lifesaver in NASH patients and as safe as in the general population.

The first study was a large prospective study and called for changing the current practice encouraging the use of statins in NASH patients as they have the highest CVD risk but dyslipidemia remains often undertreated. The second was a meta-analysis including two trials with a high risk of bias and a small number of participants.

Table 2 shows the studies used in the review.

**Table 2:** Table of studies used in review NAFLD - Nonalcoholic fatty liver disease, NASH - Non-alcoholic steatohepatitis, IR - Insulin resistance, MS - Metabolic syndrome, NLRP3 - Nucleotide-binding oligomerization domain, leucine rich repeat and pyrin domain containing protein 3, iNOS - Inducible nitric oxide synthase, eNOS - Endothelial nitric oxide synthase, HSC - Hepatic stellate cells, SMV - Simvastatin, Ras/ERK - Ras extracellular signal‑regulated kinase, RAC1 -  Ras-related C3 botulinum toxin substrate 1, FAO - Fatty acid oxidation, PPARα - Peroxisomal proliferator-activated receptor alpha, PON1 - Paraoxonase 1, CLSs - Crown-like structures, KCs - kupffer cells, EGF - Epidermal growth factor, VEGF - Vascular endothelial growth factor, PDGF - Platelet-derived growth factor, HCC - Hepatocellular carcinoma, PH - Portal hypertension, LSEC - liver sinusoidal endothelial cell, CVD - Cardiovascular disease

Author	YEAR of publication	TOPIC	ARTICLE	JOURNAL	TYPE OF STUDY	OUTCOME
Schwenger KJ,et al [3]	2014	Pathogenesis of NAFLD/NASH	Clinical approaches to non-alcoholic fatty liver disease.	World J Gastroenterol.	Review	NAFLD is associated with obesity, IR, MS. Liver biopsy confirms fibrosis and primary care is controlling the risk factors.
George N Ioannou,et al [23]	2017	Pathogenesis of NAFLD/NASH	Cholesterol crystallization within hepatocyte lipid droplets and its role in murine NASH	J Lipid Res.	Animal study	Cholesterol crystals in steatotic hepatocyte activates NLRP3 inflammasomes and kupffer cells (KCs) forming “crown-like structures” (CLSs) the results into chronic inflammation and fibrosis
Wei Wang et al, [19]	2013	Pathogenesis of NAFLD/NASH	Simvastatin ameliorates liver fibrosis via mediating nitric oxide synthase in rats with non-alcoholic steatohepatitis-related liver fibrosis	PLoS One	Animal study	Simvastatin improve NASH related fibrosis by decreasing iNOS, increasing eNOS and inhibiting HSC activation
Giovanni Musso ^ et al, [24]^	2016	Pathogenesis of NAFLD/NASH	Non-alcoholic steatohepatitis: emerging molecular targets and therapeutic strategies	Nat Rev Drug Discov	Review	There is a need for effective NASH therapy targeting molecular processes causing inflammation and fibrosis in advance stages of the disease
Robert Schierwagen et al. [17]	2016	Pleiotropic effects of statins	Statins improve NASH via inhibition of RhoA and Ras	Am J Physiol Gastrointest Liver Physiol	Animal study	SMV decreased inflammation and fibrosis through inhibition of Ras/ERK and RhoA/Rho kinase signaling pathways but RAC1 inhibition had no effect
Han-Sol Park et al, [12]	2016	Pleiotropic effects of statins	Statins Increase mitochondrial and peroxisomal fatty acid oxidation in the liver and prevent non-alcoholic steatohepatitis in mice	Diabetes Metab J.	Animal study	Statin treatment decreases both hepatic steatosis and steatohepatitis by increase in hepatic mitochondrial and peroxisomal FAO via induction of PPARα and target genes.
Mircea Vasile Milaciu et al, [22]	2019	Pleiotropic effects of statins	Paraoxonase-1 serum concentration and PON1 gene polymorphisms: relationship with non-alcoholic fatty liver disease	J Clin Med	Analytical, observational, prospective, transversal, and case-control type	NAFLD is linked to decreased PON1 serum concentration. These findings help new studies in finding non-invasive ways to diagnose NAFLD and NASH without the liver biopsy.
George N Ioannou.et al, [18]	2015	Pleiotropic effects of statins	Cholesterol-lowering drugs cause dissolution of cholesterol crystals and disperse kupffer cell crown-like structures during resolution of NASH	J Lipid Res	Animal study	Treatment with ezetimibe and atorvastatin causes resolution of fibrotic NASH, hepatic cholesterol crystals and CLSs in mouse models
Keisuke Yokohama [13]	2016	Pleiotropic effects of statins	Rosuvastatin as a potential preventive drug for the development of hepatocellular carcinoma associated with non-alcoholic fatty liver disease in mice	Int J Mol Med.	Animal study	Rosuvastatin inhibited expression of vascular proliferative factors including EGF, VEGF and PDGF may have an antitumor effect, in addition to its antihyperlipidemic effect, against HCC associated with NASH
Miren Bravo et al, [27]	2019	Pleiotropic effects of statins	Restoration of liver sinusoidal cell phenotypes by statins improves portal hypertension and histology in rats with NASH	Sci Rep	Animal study	Statins improved NASH histology as well as PH along with recovering sinusoidal endothelial function by restoring a healthy LSEC and HSC phenotype
Lee-Won Chong et al, [10]	2015	Pleiotropic effects of statins	Fluvastatin attenuates hepatic steatosis-induced fibrogenesis in rats through inhibiting paracrine effect of hepatocyte on hepatic stellate cells	BMC Gastroenterol	Animal study	Fluvastatin prevents steatosis-induced HSC activation and hepatic fibrogenesis by decreasing inflammation and oxidative stress.
Wei Wang et al, [19]	2013	Pleiotropic effects of statins	Simvastatin ameliorates liver fibrosis via mediating nitric oxide synthase in rats with non-alcoholic steatohepatitis-related liver fibrosis	PLoS One .	Animal study	Simvastatin improves NASH-related fibrosis by increasing the expression of eNOS, decreasing the expression of iNOS, and inhibiting the activation of HSC.
Vasilios G Athyros et al, [28]	2018	Statins in protective role	Statins: an under-appreciated asset for the prevention and the treatment of NAFLD or NASH and the related cardiovascular risk	Curr Vasc Pharmacol	Review	Statins have a protective effect in the treatment of NASH, significantly reduce CVD morbidity and mortality and appear safe in NASH patients.
Michael Doumas et al. [29]	2018	Statins in protective role	The role of statins in the management of nonalcoholic fatty liver disease	Curr Pharm Des	Review	Statins (specifically atorvastatin) reduce the CVD and are protective against hepatocellular carcinoma (HCC) related to NAFLD/NASH and as safe to use in NASH patients.
Konstantinos Kargiotis et al. [30]	2015	Statins in protective role	Resolution of non-alcoholic steatohepatitis by rosuvastatin monotherapy in patients with metabolic syndrome	World J Gastroenterol	Randomized Controlled Trial	Rosuvastatin monotherapy could improve NASH and completely resolves MS within 12 mo
Fernando Bril et al. [31]	2017	Statins and liver safety	Liver safety of statins in prediabetes or T2DM and nonalcoholic steatohepatitis: post hoc analysis of a randomized trial	J Clin Endocrinol Metab .	Randomized Controlled Trial	Statins can be safely used in patients with NASH
Layli Eslami et al. [32]	2013	Statins and liver safety	Statins for non-alcoholic fatty liver disease and non-alcoholic steatohepatitis	Cochrane Database Syst Rev	Review/Meta-analysis	Statins may improve serum aminotransferase levels as well as ultrasound findings.
Vasilios G Athyros et al, [28]	2018	Statins and liver safety	Statins: an under-appreciated asset for the prevention and the treatment of NAFLD or NASH and the related cardiovascular risk	Curr Vasc Pharmacol	Review	Statins have a protective effect in treatment of NASH, significantly reduce CVD morbidity and mortality and appear safe in NASH patients.
Michael Doumas et al. [29]	2018	Statins and liver safety	The role of statins in the management of nonalcoholic fatty liver disease	Curr Pharm Des	Review	Statins (specifically atorvastatin) reduce the CVD and are protective against hepatocellular carcinoma (HCC) related to NAFLD/NASH and as safe to use in NASH patients.

Limitations and considerations

More than 90% of studies included in this review are animal studies so in the future, there is a need for larger high-quality human clinical trials to establish clinical care. We also did not consider studies with other lipid-lowering agents, insulin resistance treatment, ursodeoxycholic acid, probiotics, and synbiotic, which have beneficial effects and could be accounted for in combination with statin therapy, but not been approved yet. This review might help answer a critical question of statins and NAFLD/NASH relation in terms of treatment but another one arises as we move further. So far, we have concluded that statins can be protective and effective in NAFLD but can statins be considered as an option for the future standard of care for NAFLD once diagnosed, and if it does then which one, as different statins have different potencies?

## Conclusions

Could statins prove to be preventive and therapeutic in NAFLD and NASH? This review proves the effectiveness of statins through their pleiotropic and antihyperlipidemic effects against NAFLD/NASH-associated inflammation and fibrosis. Statins achieve this goal through actions on different target points whether its RhoA and Ras inhibition, increasing PPARα expression, decreasing PON1 levels, preventing LSEC dedifferentiation and HSC activation, or resolving KCs crown-like structures. Moreover, they also improve the NASH-associated adverse outcomes like MS, CVD morbidity and mortality, and HCC prevention and are found to be safe. This study's results have taken the drug closer to resolving the question, setting a course for future researchers to follow and encouraging clinicians to use statins as a routine care for NAFLD/NASH. If confirmed further with larger human clinical trials, it might prove to be a promising drug in the treatment of NASH, which is still an unresolved mystery.
